# Serum Paraoxonase-1 Activity and the Risk of Prostate Cancer Recurrence in Patients Treated with Radiotherapy

**DOI:** 10.3390/antiox11020346

**Published:** 2022-02-10

**Authors:** Milosz Jasinski, Dorota Olszewska-Slonina

**Affiliations:** 1 Department of Urology, Institute of Oncology, Romanowskiej 2, 85-796 Bydgoszcz, Poland; 2 Department of Pathobiochemistry and Clinical Chemistry, Collegium Medicum of Nicolaus Copernicus University, M. Curie-Skłodowskiej 9, 85-094 Bydgoszcz, Poland; dorolsze@cm.umk.pl

**Keywords:** paraoxonase-1, prostate cancer, radiotherapy, recurrence

## Abstract

The antioxidant paraoxonase-1 (PON1) may be involved in the response to radiation-induced oxidative stress and possibly prevent cell apoptosis. The correlation of PON1 with the risk of cancer recurrence after radiotherapy (RT) is not yet explored. We investigated changes in the activity of PON1 in patients with prostate cancer (PCa) undergoing RT, and the relation of PON1 activity to the risk of recurrence after RT. We included 56 men with PCa. Blood samples were obtained before irradiation and after the completion of RT. Patients were followed for an average of 51.2 months. Each case of biochemical recurrence was confirmed with biopsy. The control group was composed of 60 healthy men. There was no significant difference in PON1 activity between the control group and patients pre-radiotherapy. Irradiation was associated with a significant decrease in PON1 activity. Patients with PCa recurrence had significantly higher serum PON1 activity than those recurrence-free, both before and after RT. PON1 activity was a predictor of PCa recurrence, with sensitivity over 80% and specificity over 64%. Our results suggest that PON1 activity may be a predictor of PCa recurrence risk after RT. Studies with a larger number of patients and longer follow-up are needed to confirm this hypothesis.

## 1. Introduction

Prostate cancer (PCa) is one of the most common forms of cancer in men worldwide, with radiotherapy (RT) being one of the main modalities used to treat it at the present time [1]. PCa cells, however, may develop resistance to radiation. This highly unpredictable mechanism renders the treatment less effective and frequently causes recurrence after radical RT—up to one-third of PCa patients experience biochemical failure after RT [2]. Despite identifying several molecular mechanisms associated with radioresistance, the ability to predict an individual PCa patient’s response to RT is still limited and based mostly on the pretreatment assessment of prostate specific antigen (PSA), tumor stage and Gleason score [3,4]. Therefore, there is a need to identify new biomarkers that could more accurately predict response to RT.

The majority of radiation-induced cell damage is caused by the indirect effect of free radicals produced in the effect of water radiolysis. These free radicals can initiate a chain of reactions that results in biological damage and further induces reactive oxygen species (ROS) [3,5]. PCa cells have a higher level of ROS compared with normal prostate cells and maintaining a balance of ROS levels in cancer is an important mechanism of radioresistance; RT also induces antioxidant defense systems, which may lead to radioresistance [6,7,8,9]. All these reasons justify the interest in the antioxidative mechanisms in the response of cancer cells to radiation-induced damage.

Paraoxonase-1 (PON1) is an antioxidant enzyme expressed in a variety of tissues but synthesized primarily in the liver. A portion is secreted into the plasma, where it is associated with high-density lipoproteins [10,11]. Oxidative stress and lipid peroxidation products play a role in oncogenesis, which may suggest that PON1 is associated with cancer [12]. There is a large inter-individual variability in serum PON1 activity, which can be partially attributed to the polymorphisms of the PON1 gene. Reduced PON1 activities have been reported in different groups of patients, including those with diabetes mellitus, cardiovascular disease and hypercholesterolemia. Furthermore, a number of epidemiological studies have investigated the associations between these polymorphisms and different malignancies, such as lung, breast, brain, ovarian and prostate cancers [11,13].

PON1, due to its antioxidative function, may be involved in response to radiation-induced damage and prevent radiation-induced cell apoptosis. The possible correlation of serum PON1 activity with the risk of cancer recurrence after RT is not yet fully explored, with few such studies described in the literature and none about PCa [11,14,15].

The aim of this study was to investigate the relation between serum PON1 activity and the relapse of prostate cancer in patients undergoing RT.

## 2. Materials and Methods

Fifty-six men (mean age 68.17 ± 6.95 years, range 53–80) undergoing RT for prostate cancer in the years 2005–2010 in the Department of Brachytherapy of the Institute of Oncology in Bydgoszcz, Poland were included in the prospective study. All patients had biopsy-proven prostate cancer (clinical stage T1-T3bN0M0). The average PSA before treatment was 13.35 ng/mL. All patients had pelvic magnetic resonance imaging before treatment, and prostate volume was measured in magnetic resonance images. Thirty-one patients received hormone therapy together with radiation treatment. The radiation schedule was external beam RT 46 Gy (Grey) in 23 fractions (2 Gy/day) combined with two 10 Gy high-dose-rate brachytherapy fractions [16].

Blood samples were obtained prior to irradiation and after completion of irradiation 7–8 weeks later. Sera were stored at −80 °C until biochemical analysis, however, not for more than two months. Serum PON1 activity was determined according to Playfer et al., modified by Sogorb et al., as the rate of hydrolysis of paraoxon at 37 °C, in a TRIS/HCl buffer (2-Amino-2-(hydroxymethyl)propane-1,3-diol/HCl buffer), pH 10.5, with CaCl_2_; activities were expressed as IU/L [17,18].

**Figure 1:**
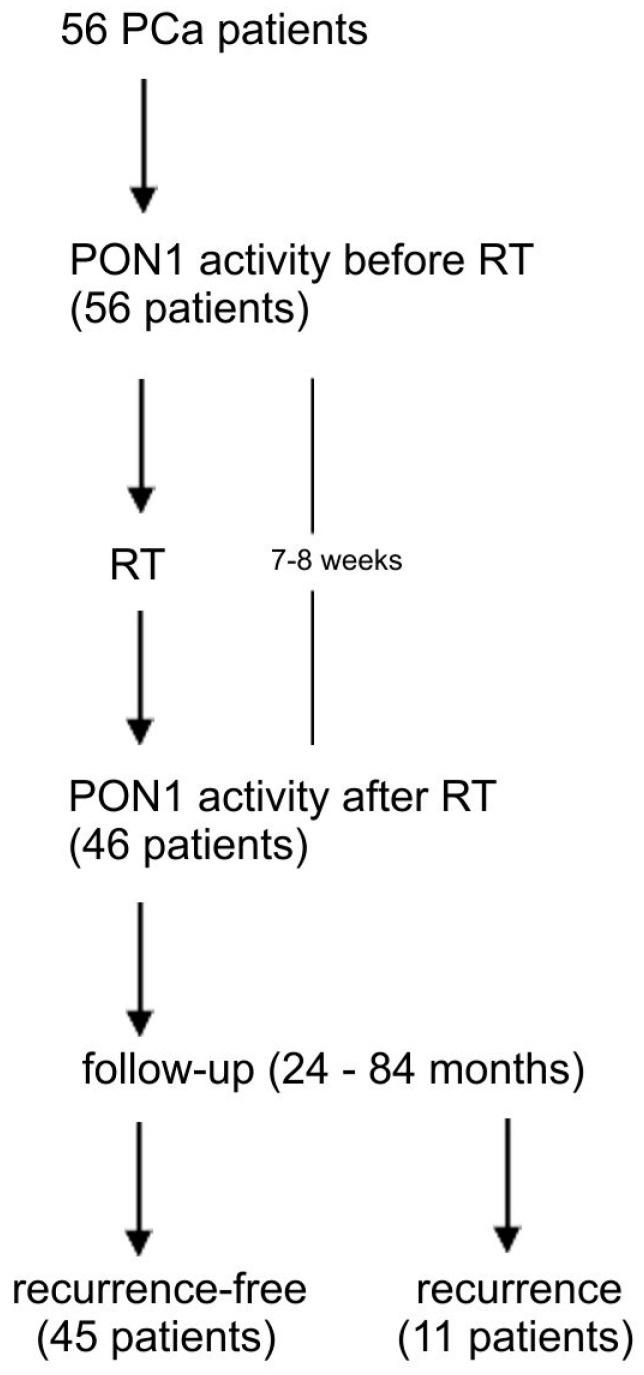
The schematic design of the study. PCa-prostate cancer, PON1-paraoxonase-1, RT-radiotherapy.

Patients were followed by PSA measurement every 3 months during the first 2 years, then every 6 months until 5 years and annually after 5 years. Biochemical recurrence was defined according to RTOG-ASTRO Phoenix criteria [19]. In every case, PCa recurrence was confirmed by prostate biopsy. The schematic design of the study is presented in Figure 1. The control group consisted of 60 healthy men (mean age 63.0 ± 13.2 years, range 56–74). They had no clinical evidence of prostate cancer nor any other malignancy.

The study was conducted in accordance with the Declaration of Helsinki. All participants (study and control group) signed written consent, provided that the anonymity of the data was guaranteed. The study was approved by the local Ethics Committee (KB/424/2005 with an annex).

Differences between any two independent groups were assessed using a Student’s *t*-test or Mann–Whitney U-test. The Wilcoxon signed-rank test was used for comparisons of dependent variables. The χ-square test was employed to evaluate differences in qualitative variables. The diagnostic accuracy of measured variables was assessed by the receiver operating characteristics (ROC) curve. The area under the curve (AUC) and 95% confidence interval values were also determined. *p* < 0.05 was considered statistically significant.

## 3. Results

### 3.1. Clinical Characteristics of PCa Patients

During the follow-up period, in 11 patients, biopsy-confirmed recurrence was diagnosed (group 1), and 45 remained recurrence-free (group 2). The main clinical characteristics of patients with and without biochemical recurrence are presented in Table 1. No significant differences were observed in age, prostate volume nor the percent of patients receiving hormonal therapy were found between the groups. Regarding TNM classification, most of the patients were classified as T1 or T2, Gleason < 7. There were no N+ or M+ patients in the classification before treatment. There were no significant differences between the groups in the risk group, according to d’Amico et al. [20].

The average follow-up length was 51.2 months (24–84). In four cases from the recurrence-free group, the follow-up time was less than 36 months; in the rest of the patients, it was at least 36 months.

### 3.2. Alterations in PON1 Activity

PON1 activity in the control group was 83.9 ± 34.5 IU/L (*n* = 60, median 76.0 IU/L, 24.3–200.6 IU/L) while in the PCa group pre-radiotherapy was 81.3 ± 41.6 IU/L (*n* = 56, median 76.1 IU/L, 20.0–169.9 IU/L). There was no significant difference in PON1 activity between the control group and PCa patients pre-radiotherapy (*p* = 0.582).

In 10 patients (two from the recurrence group and eight from the recurrence-free group), PON1 activity was measured only before radiotherapy. RT was associated with a significant decrease in PON1 activity (*p* = 0.01) (Table 2).

A decrease in PON1 activity (activity after RT lower than the one before RT) was observed in 33 of 46 cases.

### 3.3. Relationship between PON1 Activity and the PCa Recurrence

When comparing patients who remained recurrence-free to those with PCa recurrence diagnosed during follow-up, there was a significant difference in PON1 activity.

Serum PON1 activities were higher in the recurrence group, and these differences were significant both before and after radiotherapy (Table 3).

**Figure 2:**
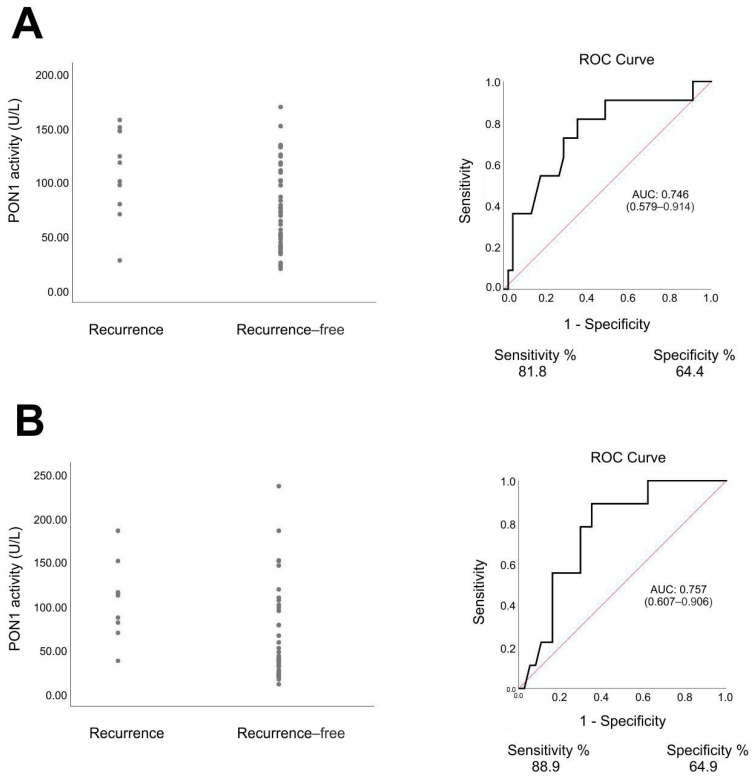
Serum paraoxonase-1 (PON1) activity and receiver operating characteristic (ROC) curves in patients with prostate cancer. (**A**) before radiotherapy, recurrence group vs. recurrence-free group, *p* = 0.012, Mann–Whitney U test. (**B**) After radiotherapy, recurrence group vs. recurrence-free group, *p* = 0.017, Mann–Whitney U test.

The statistical analysis identified PON1 as the parameter that discriminated between PCa recurrence group and recurrence-free group. ROC analysis of this parameter showed that PON1 activity allowed identification of patients in whom biochemical recurrence of PCa was later diagnosed with an AUC value of 0.746 (sensitivity 81.8%, specificity 64.4%) for the value before RT and AUC value of 0.757 (sensitivity 88.9%, specificity 64.9%) for the value after RT (Figure 2). RT-radiotherapy, SD-standard deviation

## 4. Discussion

PON1 is one of a family of three enzymes, called PON1, PON2 and PON3, which degrade lipid peroxides in circulating lipoproteins and in the cytoplasmic and intracellular organelle membranes of cells [21]. These enzymes are linked to mitochondria-associated membranes, which modulate mitochondrial metabolism and prevent apoptosis. There are variations of serum PON1 activity in the population. One source of the variability is the polymorphism in the coding and in the promoter region of the PON1 gene. There are also exogenous factors modulating PON1 activity and levels of expression, such as certain drugs, dietary factors, and lifestyle factors. Costa et al., in their review, described several factors responsible for the differences in PON1 activity in populations [22]. There are numerous studies linking PON1 with the risk of cancer, including PCa [12,13,23,24,25]. Several studies have also reported decreased serum PON1 activity in cancer patients [14]. While Bedir et al. observed lower levels of PON1 in patients diagnosed with PCa, but before treatment, than in healthy population, the exact mechanism involved in the decrease in PON1 activity in PCa patients is still unclear [26]. In our study, we did not observe such a difference between PCa patients before treatment and the control group. We did, however, find a significant decrease in PON1 activity after RT. This phenomenon is not surprising—RT leads to elevated oxidative stress and degrades lipid peroxides, PON1 binds covalently to lipid molecules leading to enzyme inactivation [27]. Hence, the result of increased oxidative stress is decreased PON1 activity. A similar decrease in serum PON1 activity after RT has also been observed in other studies [14,15].

The most noteworthy result of this study is the significant difference in PON1 activity between the group of patients who were diagnosed with PCa recurrence and those who remained recurrence-free. As far as we know, this is the first study to investigate associations of the PON1 activity with the response to RT in PCa patients. Interestingly, PON1 activity, both before and after RT, was the only statistically significant difference between recurrence and recurrence-free groups in this study. The AUC of the ROC curves of PON1 activity in the distinction between patients with and without PCa recurrence after RT were 0.746 and 0.757, which are favorable in comparison with prediction models based on clinical variables and imaging features [28]. Our study suggests that the determination of PON1 activity may be a potential marker in the prediction of PCa response to RT. If an 80 U/L cut-off value of PON1 activity before RT was used, it could predict PCa recurrence in the studied group with almost 82% sensitivity and over 64% specificity. Rodriguez-Tomas et al., in their study on lung cancer, made a somewhat similar observation—patients who presented a complete response after RT had lower PON1 serum concentrations than those who presented a partial response or did not respond to RT [14]. While we did not measure the PON1 serum concentration in our study, it could be worth investigating if there is such a relation in PCa patients.

Available literature provides no explanation of the reasons for this observation, nor can they be deduced from the present study. We may, however, suggest two possible mechanisms based on the role of PON1 and the metabolic changes induced by the cancer-related increased oxidative stress, which are not mutually exclusive.

First, the antioxidative role of PON1 may partially protect PCa cells from radiation-induced damage, and this protective effect may be stronger in patients with higher serum PON1 concentration and activity. One of the mechanisms of radiation-induced cell damage involves damage of mitochondrial integrity, leading to mitochondrial dysfunction, increased oxidative stress and cell death. Radiation increases mitochondrial ROS production by inducing secondary ROS generation via electron leakage from the mitochondrial electron transport chain complexes [29,30,31]. On the other hand, overexpression of the enzymes from the PON family prevents mitochondrial dysfunction—they are linked to mitochondrial membranes, modulate mitochondrial metabolism and prevent apoptosis [32,33,34]. It is also suspected that cancer cells can scavenge serum PON1 and take advantage of its antioxidative effects [35,36,37].

Second, a decrease in serum PON1 activity may be a marker of the radiosensitivity of PCa. The response of cells to radiation strongly depends on oxygen, with hypoxia reported as one of the mechanisms leading to radioresistance [38]. Milosevic et al. found that local hypoxia was associated with a higher risk of PCa recurrence after RT [39]. Hypoxia has a significant effect on cell metabolism—a low basal ROS level is maintained in hypoxic cells by reduced mitochondrial ROS production and promotion of ROS detoxification processes [40,41]. This may lead to a hypothesis that the mechanisms involved in hypoxia-induced radioresistance also lead to a decrease in cancer-related increased oxidative stress and thus partially prevent the decrease of serum PON1 activity.

What is also important, PON1 activity was higher in the recurrence group even before the treatment. Therefore, it may be a useful marker in clinical decision making. There is a large group of PCa patients with localized disease who may be treated either by radical prostatectomy or RT, without a clear indication for one of these modalities. Knowing who may be potentially at greater risk of PCa recurrence after RT could result in offering some of these patients the alternative treatment method (radical prostatectomy).

This study has several limitations. Due to the number of patients studied, the recurrence group is small. The length of follow-up may not be enough to observe every recurrence in the case of PCa, and there is a risk that some late recurrences were omitted. Our results should be validated and confirmed in larger, preferably multi-center studies with longer follow-up. Further studies would also be essential to determine the cut-off value of PON1 activity, predicting the risk of PCa recurrence after RT and the possible relation of PON1 concentration with the risk of recurrence, especially since such relations were reported for lung and breast cancers [11,14]. This is the first study investigating PON1 activity in relation to the risk of PCa recurrence after radiotherapy; therefore, information available in the literature on this subject is scarce, limiting the quality of the discussion.

## 5. Conclusions

The results of the present study suggest the usefulness of investigating alterations in oxidative stress during RT in PCa patients. We conclude that PCa patients presented a significant decrease in serum PON1 activity during RT. Moreover, we observed significantly higher PON1 activity in patients who experienced PCa recurrence after RT. Due to the small number of patients studied, our results must be considered preliminary, but they suggest that the determination of PON1 activity might be a valuable tool for the prediction of PCa recurrence after RT, possibly more accurate than prediction models based on clinical variables. Further studies are necessary to evaluate the usefulness of PON1 activity in this clinical situation and to determine the cut-off values.

## Figures and Tables

**Table 1:** Clinical and demographic characteristics of prostate cancer patients.

Clinical and Demographic Characteristics	PCa Recurrence	PCa Recurrence-Free	*p*-Value
*n*	11	45	
Age (mean ± SD) [y]	68.09 ± 6.16	68.20 ± 7.20	*p* = 0.96 *
TNM			*p* = 0.26
T1	9.1% (*n* = 1)	24.4% (*n* = 11)	
T2	81.8% (*n* = 9)	66.7% (*n* = 30)	
T3	9.1% (*n* = 1)	8.9% (*n* = 4)	
Gleason score			*p* = 0.42
<7	63.6% (*n* = 7)	75.6% (*n* = 34)	
7	18.2% (*n* = 2)	13.3% (*n* = 6)	
>7	18.2% (*n* = 2)	11.1% (*n* = 5)	
PSA (mean ± SD) [ng/mL]	19.7 ± 26.16	11.8 ± 8.35	*p* = 0.85 *
Risk group			*p* = 0.82
Low	36.4% (*n* = 4)	33.3% (*n* = 15)	
Intermediate	36.4% (*n* = 4)	44.4% (*n* = 20)	
High	27.2% (*n* = 3)	22.3% (*n* = 10)	
Prostate volume (mean ± SD) [mL]	37.9 ± 15.1	33.2 ± 14.3	*p* = 0.37 **
Hormone therapy	45% (*n* = 5)	58% (*n* = 26)	*p* = 0.46

PCa-prostate cancer, y-year. Statistical analyses were performed by the χ-square test; * Student’s *t*-test; ** Mann–Whitney U test.

**Table 2:** Serum paraoxonase-1 (PON1) activity in prostate cancer patients before and after radiotherapy (RT).

*n* = 46	PON1 before RT [IU/L]	PON1 after RT [IU/L]	Wilcoxon Test
Mean ± SD	83.5 ± 43.2	74.5 ± 55.0	*p* = 0.010
Median (range)	75.0 (20.0–169.9)	55.6 (11.2–236.8)	

**Table 3:** Serum paraoxonase-1 (PON1) activity before and after radiotherapy in prostate cancer patients who experienced recurrence after treatment and in patients who remained recurrence-free.

Serum PON1 Activity	Recurrence	Recurrence-Free	Mann–Whitney Test
*n*	11	45	
PON1 before RT [IU/L] mean ± SD	111.3 ± 40.7	74.0 ± 38.9	*p* = 0.012
Median (range)	118.4 (27.8–158.0)	69.2 (20.0–169.9)	
*n*	9	37	
PON1 after RT [IU/L] mean ± SD	106.4 ± 44.2	66.7 ± 53.8	*p* = 0.017
Median (range)	112.4 (37.9–186.1)	40.3 (11.2–236.8)	

## Data Availability

Data generated or analyzed during this study are included in this article. More detailed data are not publicly available due to their containing information that could compromise the privacy of patients. Further inquiries can be directed to the corresponding author.
